# Distal Junctional Failure: A Feared Complication of Multilevel Posterior Spinal Fusions

**DOI:** 10.3390/jcm13174981

**Published:** 2024-08-23

**Authors:** Soufiane Ghailane, Houssam Bouloussa, Manuel Fernandes Marques, Jean-Etienne Castelain, Vincent Challier, Matthieu Campana, Clément Jacquemin, Jean-Marc Vital, Olivier Gille

**Affiliations:** 1 Department of Spinal Surgery Unit, Hôpital Privé Francheville, 24000 Périgueux, France; drcastelain@hpdf.fr (J.-E.C.); challier.vincent@gmail.com (V.C.); drcampana@hpdf.fr (M.C.);; 2 Department of Orthopaedic Surgery, University of Missouri-Kansas City, 2301 Holmes Street, Kansas City, MO 64108, USA; houssam.bouloussa@gmail.com; 3 Unidade Local de Saúde do Litoral Alentejano, Serviço de Ortopedia, Monte do Gilbardinho, 7540-230 Santiago do Cacém, Portugal; manuelfernandesmarques@gmail.com; 4 Department of Spinal Surgery Unit 1, Université de Bordeaux, Bordeaux University Hospital, C.H.U Tripode Pellegrin, Place Amélie Raba Léon, 33076 Bordeaux, France; vital.jean-marc@wanadoo.fr (J.-M.V.); olivier.gille@chu-bordeaux.fr (O.G.)

**Keywords:** distal junctional failure, spinal deformity, hardware failure, thoraco–lumbar instrumentation, revision surgery, mechanical complications

## Abstract

**Objectives:** Distal junctional failure (DJF) is less commonly described than proximal junctional failure following posterior spinal fusion, and particularly adult spinal deformity (ASD) surgery. We describe a case series of patients with DJF, taking into account sagittal spinopelvic alignment, and suggest potential risk factors in light of the current literature. **Methods:** We performed a single-center, retrospective review of posterior spinal fusion patients with DJF who underwent subsequent revision surgery between June 2009 and January 2019. Demographics and surgical details were collected. Radiographical measurements included the following: preoperative and postoperative sagittal and coronal alignment for each index or revision surgery. The upper-instrumented vertebra (UIV), lower instrumented vertebra (LIV), and fusion length were recorded. **Results:** Nineteen cases (64.7 ± 13.5 years, 12 women, seven men) were included. The mean follow-up was 4.7 ± 2.4 years. The number of instrumented levels was 6.79 ± 2.97. Among the patients, 84.2% (n = 16) presented at least one known DJF risk factor. LIV was frequently L5 (n = 10) or S1 (n = 2). Six patients had an initial circumferential fusion at the distal end. Initial DJFs were vertebral fracture distal to the fusion (n = 5), screw pull-out (n = 9), spinal stenosis (n = 4), instability (n = 4), and one early DJK. The distal mechanical complications after a first revision included screw pull-out (n = 4), screw fracture (n = 3), non-union (n = 2), and an iatrogenic spondylolisthesis. **Conclusions:** In this case series, insufficient sagittal balance restoration, female gender, osteoporosis, L5 or S1 LIV in long constructs were associated with DJF. Restoring spinal balance and circumferentially fusing the base of constructs represent key steps to maintain correction and prevent revisions.

## 1. Introduction

The treatment of adult spinal deformity (ASD) is known for the frequent necessity to perform complex surgeries that usually require long segment fusions to correct and stabilize the deformed spine. These procedures tend to be more and more frequent due to the growing demand of spinal surgery. Aging populations with an increased life expectancy strongly desire a higher quality of life. Older patients pose several challenges and complications that are typically not present in pediatric or adolescent spinal deformity surgery. Numerous medical and surgical complications occur during or after these procedures. Yet, the mechanism behind the development of junctional spinal disorders, especially at the lower end of constructs, still remains poorly understood. The presence of osteoporosis, multiple medical comorbidities, smoking, neurological disorders responsible for the deformity (such as Parkinson’s disease), an aging and rigid spine with ligamentous degeneration, hip osteoarthritis, muscle fatty infiltration, and a severe stiffness mismatch between the bone and spinal implants all contribute to the challenge posed by the surgical treatment of patients with ASD [1]. Mechanical complications related to long constructs represent the most frequently reported complications in ASD surgery (up to 30%) [2].

Junctional disorders above the construct, also known as proximal junctional kyphosis (PJK) or proximal junctional failure (PJF), represent a much dreaded, well-described, and frequent complication, while the majority of cases remain asymptomatic. Degeneration can also arise distally to the fusion in the form of distal junctional kyphosis (DJK) or failure (DJF) [3]. While PJK is usually a more progressive and sub-clinical entity which occurs in 6.8% to 30% of cases, DJF is far less frequent, much more acute and spectacular in its presentation. Distal junction failure can also appear in the form of different clinical entities according to Arlet and Aebi [1], which can be any combination of progressive loss of lumbar lordosis, loss of disc height with disc degeneration, spinal stenosis, segmental instability, acute wedging in the distal junctional disc, fracture of the lower instrumented vertebra, and failure of the instrumentation at the most distal level (most frequently at L5).

Several strategies to prevent distal junctional failure in ASD surgery were proposed, such as avoiding ending a long construct at L5 or at S1 without protecting it with pelvic fixation [4], avoiding overcorrection (>50%), and incorporating the first lordotic disc into the fusion construct. Yet, there is a lack of evidence in the literature to support these. Our goal was to describe a case series of patients with a multilevel posterior lumbar spinal fusion requiring revision surgery for DJF, taking into account sagittal spinopelvic alignment, to identify potential clinical and radiographic risk factors, and finally propose prevention and revision strategies in light of the existing literature.

## 2. Methods

### 2.1. Study Design and Population

From June 2009 to January 2019, the data from 19 patients treated for DJF/DJK were retrospectively collected and analyzed in a single center. A local ethics committee approved this study. Inclusion criteria were (1) degenerative spinal deformity and/or (2) multilevel posterior spinal fusion, (3) requiring operative revision, and (4) six-month minimum follow-up. Demographic data were collected, such as age, gender, BMI, symptoms including abnormal neurological findings previous to treatment, number of revision cases, and relevant comorbidities, such as osteoporosis, smoking status, Parkinson’s disease, alcohol abuse. Surgical data were also collected, including the surgical approach, type of instrumentation, presence of osteotomy and interbody fusion at the most caudal level, perioperative complications, and the need for revision surgery. All charts from all cases and complications were carefully analyzed and discussed with the operator in order to identify a potential cause for the failure. Eventually, a consensus was reached for each case by three attending surgeons. A radiographic analysis was conducted on preoperative standing X-rays (index procedure), postoperative standing X-ray (within 1 week of the index procedure), preoperative standing X-ray (before each revision), postoperative standing X-ray (within 1 week of each revision), and at final follow-up. The measured radiographic parameters were as follows: spinopelvic parameters (pelvic incidence, PI, lumbar lordosis, LL, sacral slope, SS, pelvic tilt, PT, PI-LL, sagittal vertical axis, SVA), lower instrumented vertebra (LIV), upper instrumented vertebra (UIV), number of fused levels, and the presence of an interbody spacer.

### 2.2. Definition of Distal Junctional Failure/Distal Junctional Kyphosis

DJK was defined as an abnormal distal junctional angle superior or equal to 10 degrees and at least 10° greater than the preoperative value [3]. The presence of both criteria was necessary to classify each event. The distal junctional angle was defined as the Cobb angle between the superior endplate of the LIV and the inferior endplate of the adjacent vertebra distal to the LIV (LIV + 1). This typically translated into loss of lumbar lordosis or disc degeneration, with loss of height.

Different types of DJF were identified: acute disc wedging distal to the instrumentation, fracture of LIV, osteoporotic fracture distal to the construct, failure of the instrumentation at the LIV, spinal stenosis, segmental instability distal to the construct [1]. In this context, instability was defined as a difference of either 5 degrees or more in motion within a fusion segment observed between flexion and extension, or a vertebral body translation of 3 mm or more [5,6].

### 2.3. Potential Risk Factors

Potential risk factors screened in this case series [7,8]:

- Older age (>65 years old);
- Osteoporosis;
- Long fusion (>4 levels);
- Fusion short of the first lordotic disc (FLD);
- Stopping the LIV at L5 or S1 without iliac fixation;
- Substance abuse/smoking;
- Hip osteoarthritis;
- Overcorrection of sagittal deformity greater than 50%;
- Sagittal imbalance.

### 2.4. Statistical Analysis

Statistical analysis was performed using IBM SPSS Statistics software version 21.0 (IBM, Armonk, NY, USA). All data were expressed in mean values ± standard deviations. Comparisons between pre- and postoperative changes were evaluated using the Mann-Whitney U test. Statistical significance was assumed at *p* values of less than 0.05.

## 3. Results

The preoperative and postoperative clinical and radiographic data are summarized in Table 1 and Table 2.

### 3.1. Population and Operative Data (Table 1)

Nineteen revision cases due to distal junctional failure after multilevel posterior spinal fusion surgery were reported. The mean age was 64.74 ± 13.55 years and the average follow-up was 4.7 ± 2.4 years. The sex ratio was 1.7:1, 12 women and seven men. There were eight cases of scoliosis or kyphoscoliosis (42.1%), two cases of trauma complicating elective spine surgery (10.5%), and five cases involving one or more degenerative spondylolisthesis (26.3%). The average number of instrumented levels was 6.79 ± 2.97. We observed that 84.2% (n = 16) of the patient sample had at least one known risk factor for mechanical complications. The most frequently observed LIV was L5 in 10 cases (52.6%), while two patients (10.5%) had a LIV at S1 without pelvic fixation. Only six patients (31.5%) had a circumferential fusion at most caudal level (TLIF only), performed during the index surgery. Reported initial DJFs were vertebral fracture distal to the fusion (n = 5), screw pull-out (n = 9), spinal stenosis (4), instability (n = 4), and one early DJK. Mechanical complications after a first revision for DJF included screw pull-out (4), screw fracture (3), non-union (2), and an iatrogenic spondylolisthesis at the distal end of the construct. Mechanical complications after a second revision for DJF included screw pull-out (2) and non-union (2). The average time to revision for DJF was 15.62 ± 17.86 months. Eight patients (42.1%) required a revision during their first postoperative year. The most frequently performed revision surgery was a distal extension of the fusion with iliac bolts. In this series, nine patients (47.3%) only required one revision. At the second revision stage, there were nine patients (47.3%). Finally, three required a third revision (15.7%). Iliac bolts were used only twice at the index procedure (10.5%), in conjunction with circumferential fusion at the distal end (L5–S1 TLIF). However, these two cases failed with failure at the iliac screws. Iliac bolts alone did not prevent failures. Indeed, they were either fractured (five times) or loosened/pulled out (six times). The use of iliac bolts was more prevalent in the revision cases: 12 cases (12/18, 66.6%) with iliac bolts at the first revision, 7 cases at the second revision (7/9, 77.7%), and 2 at the third revision (2/3, 66.6%). Success or failure can be defined by the need for subsequent revisions. In this case, regarding the outcomes of circumferential fusion (TLIF, ALIF, or LLIF) associated with pelvic fixation, this association was employed 12 times and resulted in mixed results (six failures and six successes). In order to prevent further revisions, the association of ALIF at L5–S1 and pelvic fixation was employed five times and provided the best results: four successes (no revisions to date) and one failure.

### 3.2. Radiographic Analysis (Table 2)

**Figure 1:**
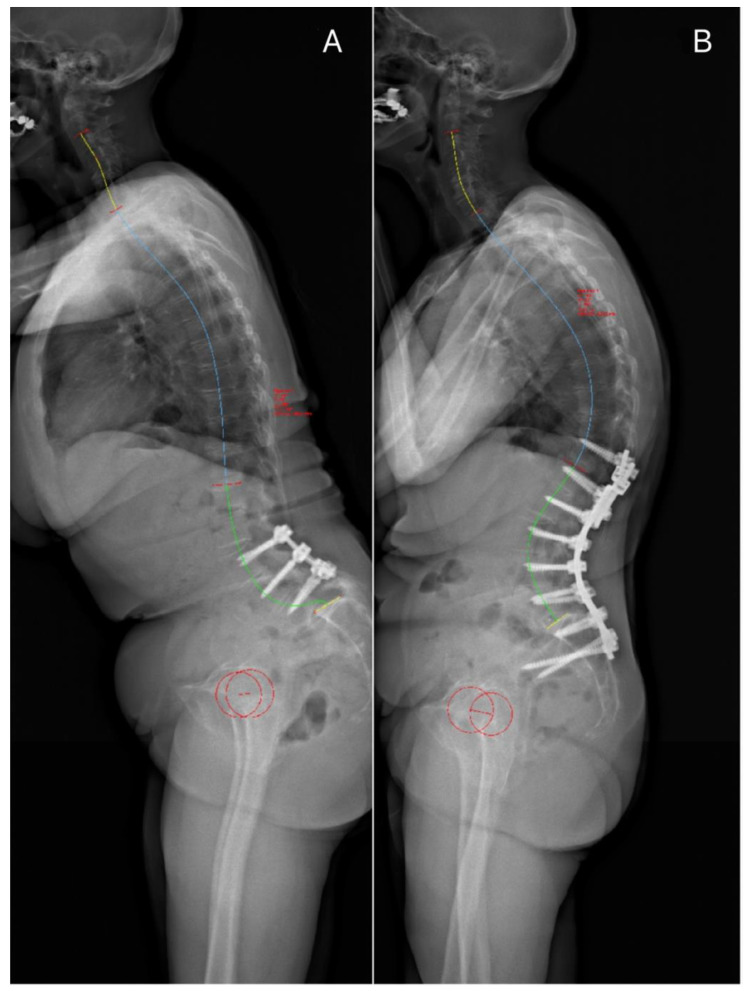
Preoperative (**A**) and postoperative (**B**) standing X-rays following revision for L5S1 dislocation (case n° 12): ((**A**) PT 43°, PI 74°, LL −26°, PI-LL 48°, SVA 199.27 mm; (**B**) PT 40°, PI 76°, LL 68°, PI-LL −9, SVA 62.13 mm).

**Figure 2:**
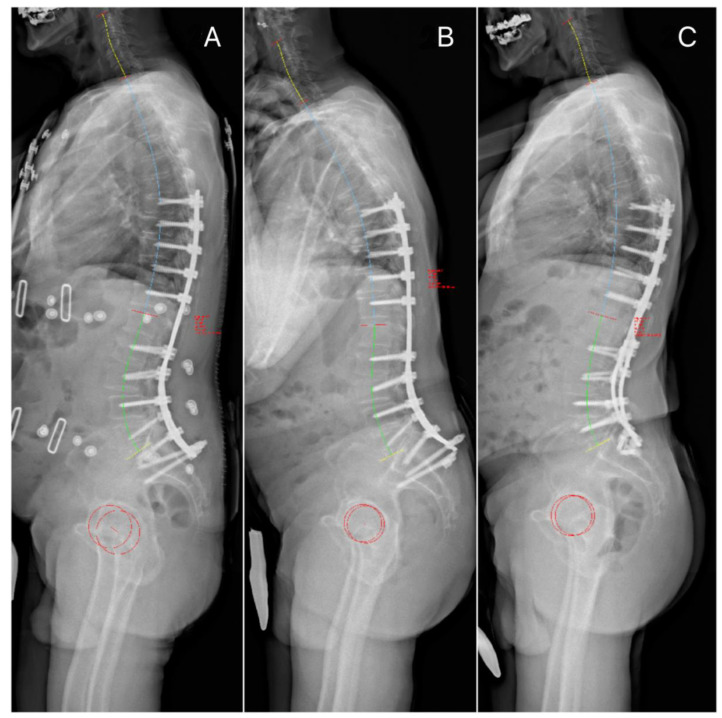
Preoperative (**B**) and postoperative (**C**) standing X-rays following revision for L5 and iliac screw pull-out. (**A**) shows the immediate postoperative (index surgery) standing X-ray before failure (case n° 17). (**A**). PT 15°, PI 53°, LL −50°, PI-LL 3, SVA 33.24 mm (**B**). PT 22°, PI 48°, LL −25°, PI-LL 23, SVA 126.29 mm (**C**). PT 19°, PI 48°, LL −44°, PI-LL 4, SVA 24.88 mm.

Despite the fact that before each surgery, most patients were sagittally malaligned (Figure 1A and Figure 2B), revision surgery partially restored the SVA (Figure 1B and Figure 2C), though this was not statistically significant (*p* > 0.05). During follow-up, the preoperative SVA increased with each complication and revision surgery. SVA correction decreased as well with each complication and revision surgery. Interestingly, each surgery tended to reduce the PI-LL mismatch, while this was statistically significant only for the index cases. Moreover, the patients revised twice had a significantly higher PI than the patients revised only once (*p* < 0.05) (Table 2). Lumbar lordosis correction increased with the number of revisions, though it did not reach statistical significance. Indeed, the average lumbar lordosis correction for patients with two failures or more was about three times higher than the average lumbar lordosis correction observed in the index case (*p* < 0.05) (Table 2).

**Figure 3:**
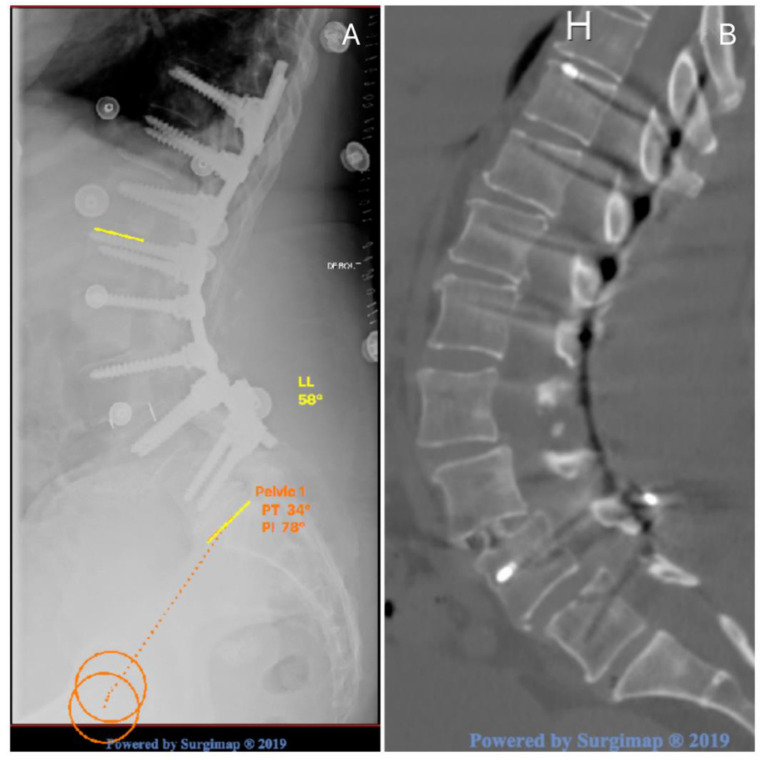
Post operative X-rays (**A**) with a stop at L5 in long construct ((**A**) PT 34°, PI 78°, PI-LL 20); with L5 superior plate fracture confirmed by CT-scan (**B**).

Figure 1, Figure 2 and Figure 3 illustrates some typical cases, and the management of DJF.

## 4. Discussion

Industrial societies have been challenged by an aging population with constantly higher functional demands [9]. As a result, every year sees a further increase in adult spinal deformity cases [2]. However, these cases are technically difficult due to advanced degenerative processes, osteoporosis, and weakened posterior elements (fatty degeneration of muscles). Pre-existing sagittal malalignment and co-existing hip and knee osteoarthritis may also add a level of complexity to these cases [10]. All these factors contribute to a higher risk of postoperative malalignment, which is a well-described predictor of hardware failure [11,12,13]. Because the vast majority of failures occur at the proximal end of spinal constructs, distal junctional failure has been relatively poorly described to this date. Obviously, a common factor in these cases is a high mechanical stress at the lower end of the construct. Our belief is that in most cases, a local or regional angular abnormality leads to postoperative malalignment, which in turn becomes the genesis of high mechanical stress at both upper and lower extremities. In order to reduce that stress, the most ergonomic and energy-efficient strategy is unconsciously or semi-consciously chosen by patients above and below the fusion (pelvic retroversion if hips joints are not osteoarthritic, thoracic hypokyphosis, knee flexion) [14]. The lower energy option is often the first chosen. When these mechanisms are overrun, the energy can be restituted in various forms (screw pull-out, fracture, dislocation, rod or screw fracture) [15].

These results suggest that DJF is usually not an isolated event. It behaves similar to a mechanical cascade that can only be stopped surgically. Indeed, the majority of our patients had more than one revision. The SVA correction tended to follow a law of diminishing returns after each mechanical complication and therefore revision. In addition, the lumbar lordosis average correction increased with each round of revision, which shows that more effective corrective osteotomies were employed after each failure. The fact that the PI was higher in patients with two revisions or more suggests that high sheer forces at the distal end of the construct increases the risk of DJF and renders revisions more challenging.

Regarding the treatment pattern, there was a general tendency to reinforce the distal end of the construct with either an interbody device for circumferential fusion or iliac bolts, or both (Table 1). If there was a TLIF at the most caudal level and failure occurred, an ALIF was performed. If the ALIF failed, it was revised with iliac bolts. Only six patients had a distal circumferential fusion at the index procedure. Retrospectively, it appears as probably too few, especially at the distal end. Interestingly, there was no ALIF as a means of circumferential fusion in the index procedure, which is the result of surgeons’ habits. Only TLIFs were used for that purpose. ALIFs were reserved for revisions. Interestingly, the combination of an ALIF at L5–S1 with pelvic fixation appeared to arrest the progression of the “revision cascade”. This may be explained by the lordotic effect of the L5–S1 ALIF cages, which in theory can more easily correct SVA since they are located at the bottom of the construct. Because of the immense constraints in long fusions and obese patients particularly, it seems retrospectively logical to protect iliac bolts with a lumbosacral fusion anteriorly. Yet, this may have been falsely considered a success due to a lack of follow-up.

Failure is not exclusively the result of instrumentation. It can also result from soft tissue damage. Indeed, the posterior tension-band was damaged in three cases in this series. It is probable that this played a role in the development of DJF. Furthermore, removing instrumentation at the distal end on a previous fusion probably resulted in failure in case 1.

In most cases, a lesson can be learnt from a failure, and this is the reason why the authors chose to share this case series with esteemed peers. Failures do not occur randomly. There are patterns of techniques and patients that lead to them. In addition, the lever arm represented by long instrumentations is usually greatest on the lower end of the constructs, which may explain the «spectacular» and early hardware failures defined as distal junctional failures. Poor understanding of the etiology of a hardware failure will ultimately lead to another disaster. Screws do not always pull-out because of poor bone density, and augmented screws with PMMA are not the sole answer for the prevention of junctional failures. Obviously, if a patient is significantly malaligned immediately postoperatively, any surgical intervention aimed at reducing the rate of junctional failure is bound to fail (screws may pull out along with their cement block or fracture the entire vertebra; fractures or dislocations occur in vertebrae peripheral to the instrumentation despite vertebroplasty at junctional levels). This vicious circle can be broken once the extremities are off-loaded, i.e., when the patient is adequately aligned. Several risk factors for DJF were described in the literature [7,8,15].

They can be separated into two main categories: factors that can be influenced by the surgeon (sagittal imbalance, overcorrection, lower instrumented vertebra selection). DJF may result in imbalance and unacceptable deformity. It is not always detectable clinically, and it is often not predictable from the analysis of preoperative radiographs [16,17]. Furthermore, overcorrection may occur in younger patients (children with adolescent idiopathic scoliosis or Scheuermann’s disease). Preoperative thoracolumbar kyphosis is a risk factor for the development of postoperative DJK. Similarly, the presence of increased kyphosis after surgery in the T10–L2 region constitutes a “risk factor” for the development of DJK [16]. The LIV selection is also critical, especially in Scheuermann’s disease. In this scenario, stopping short of the FLD or not including the stable sagittal vertebrae (SSV) or a high residual LIV plumb line will lead to a higher risk of DJK and DJF [1]. In the case of DJK, these patients should receive more attention and be scheduled for continuous follow-up in the first postoperative years [18]. Another group of DJF risks factors comprise those that cannot be influenced by the surgeon (high BMI, osteopenia/osteoporosis, older age, substance abuse, multiple previous surgeries, and concomitant hip osteoarthritis). Whenever they are modifiable, adequate treatment should be initiated in order to mitigate the risk of postoperative DJF.

Multiple prevention tools are available to surgeons. Matching lumbar lordosis to pelvic incidence and respecting the global sagittal alignment is one of them [19,20]. Another issue is that lordosis mostly affects the L4–S1 segment rather than the L1–L4 segment. Finally, respecting the posterior tension band, as it was shown in PJK or PJF prevention [8], including the first lordotic disc in a thoracic kyphosis correction [1], choosing the right instrumentation stiffness adapted to the anticipated post-correction spine stiffness (rod diameter, metal type) [21], creating a “soft-landing” with sublaminar bands [22] or hooks at the extremities of fusion constructs (as can be used for PJK and PJF prevention) [23] are other commonly described tips and tricks, though there is no consensus or even high evidence backing their use. Because most of these factors were described in the setting of the research on PJK and PJF, using these principles in a similar manner at the distal end of constructs may not necessarily prove to be adequate in the future. Further specific research is needed for DJF.

In addition, it is wise to analyze the lumbosacral “base” of a construct following a PJF event. Indeed, our belief is that the same reasons (sagittal malalignment, often between L4 and S1) can alternatively lead to PJF or DJF. This has yet to be demonstrated.

Conflicting evidence exists for the choice of the LIV to prevent DJK. Yang et al. [24] showed that stopping at or below the SSV nearly eliminates the risk of DJK in the case of AIS with selective fusions. Yet, this was achieved at the expense of incorporating additional fusion levels. What typically happens at L5–S1 post-fusion is another controversial topic. Cho et al. reported that subsequent advanced L5–S1 disc degeneration occurred in 58% of the patients whose fusion ended at L5 [25]. Again, these studies focus on DJK and typically do not address DJF. Fusing on S1 would eliminate the possibility of L5–S1 disc degeneration and loss of lordosis, but it is also associated with a higher rate of pseudarthrosis. Yasuda et al. recommended spinopelvic fixation using iliac bolts for long corrective fusions in the setting of adult spinal deformity surgery, given the high failure rate in patients with long fusions when stopping at L5 or S1 in their series [4,25]. The current work emphasizes the importance of lower lumbar lordosis correction (L4–S1) with anterior support (tall interbody devices with significant built-in lordosis) to increase the fusion rates and achieve satisfactory postoperative balance. Indeed, the best chances of reaching optimal sagittal alignment seems to lie below the instrumentation and not above it. In the case of multiple revisions, it is obvious that addressing only the symptom (for example toggling screw treated with percutaneous vertebroplasty in the pelvis or vertebra) will not arrest the underlying mechanical issue. Avoiding the repetition of similar mechanical mistakes, preoperative surgical planning (stiffness, alignment, screw type, cementation), preparing the patient to control non-surgeon-dependent DJF risk factors (BMI, T-score, smoking cessation, osteoporosis treatment, evaluation and treatment of hip osteoporosis) [26] are not the unique keys to success but help avoid taking the highway to failure. More recently, McDonnell et al. demonstrated in a systematic review on DJF following ASD surgery that stopping at L5 was risk factor for developing DJF postoperatively and that there could be a protective effect of anterior–posterior fusions on postoperative DJF [27]. In a subsequent study, they showed that performing a pedicle subtraction osteotomy, undercorrecting lumbar lordosis and sagittal vertical axis, are significantly associated with postoperative DJF [28]. The role of sagittal balance was again shown to be of importance by Montanari et al. in their retrospective case–control cohort study of posterior spinal fusions. Indeed, they found an association between DJF and age over 40 years, as well as a lack of restoration of PT, PI-LL, and T1 pelvic angle (TPA) [29].

**Figure 4:**
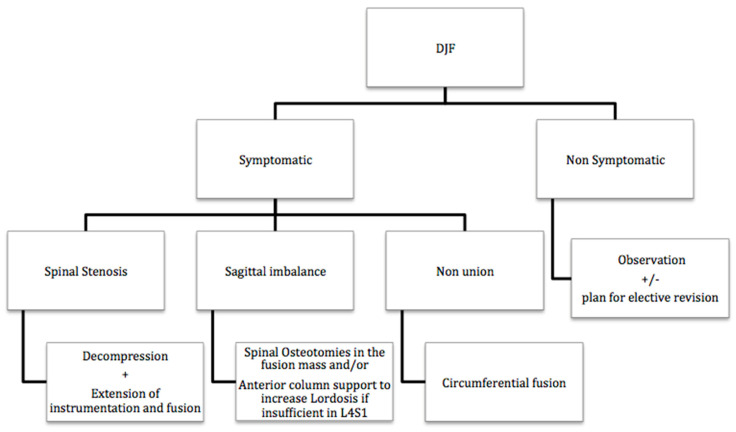
Proposed algorithm for the management of DJF based on clinical symptoms and associated diagnoses (spinal stenosis, sagittal imbalance, non-union).

A simple algorithm for DJF management is proposed in Figure 4.

### Limitations of This Study

The number of patients is low and reflects failures of multilevel posterior lumbar fusion surgery in a single center over a 10-year period. Given the fact that DJF is a rare event, this study is most likely too under-powered to detect new risk factors. During the study period, there were approximately 7500 spine fusions performed in our department. The focus was on DJF cases that required a revision surgery. The global incidence was therefore 0.25% for all fusions performed, which includes non-ASD surgeries. A multi-center and controlled prospective study would have overcome this obstacle. However, surgeons are usually reluctant to report their worst complications. It was therefore decided to proceed with a single-center study only and raise awareness about the cascade of complications generated by DJF. The retrospective nature of this study and the absence of controls also has inherent limitations. Yet, there is great interest in defining the patterns between two different types of DJF: those that are “cured” and those that recur.

## 5. Conclusions

The current data analysis suggests that female gender, osteoporosis, stopping at L5 or at S1 without iliac fixation in long constructs with UIV proximal to L1 represent high risks of DJF. Insufficient sagittal alignment restoration also probably represents a high risk of DJF and revision surgery. The association of an anterior lumbosacral fusion with pelvic fixation appeared to decrease the risk of the recurrence of DJF.

The main goals of the treatment of DJF are to restore spinal balance, through a posterior approach (hardware revision, correction, treating non-union) and to obtain a solid anterior fusion with a wide and lordotic spacer (either through a traditional anterior retroperitoneal approach for an anterior lumbar interbody fusion, ALIF, or a minimally invasive lateral approach such as a lateral lumbar interbody fusion, LLIF).

This series shows that this type of circumferential fusions may help maintain surgical correction, increase the rate of fusion, and reduce the overall longer-term mechanical complications. The efficacy of PSO should be balanced with its high risk of complications reported in the literature. It is to be avoided as a treatment for sagittal imbalance below a long fusion with DJF. Yet, it may still be an option in the case of an iatrogenic flatback and DJF.

## Figures and Tables

**Table 1:** Main demographic and surgical data from 19 cases of distal junctional failure.

N	Age	Sex	Diagnosis/ Prior History	Index Surgery	Levels	IBD	Sagittal Imbalance (PI-LL Mismatch)	RF	Potential Cause	1st Failure	1st Revision	2nd Failure	2nd Revision	**3rd Failure**	**3rd Revision**
1	73	F	T10 fracture and prior L2–L4 fusion	T10–L1 fusion, L2–L4 removal, L1–L2 decompression	4	No	N/A	Osteoporosis	L5 stop	L5 fracture	L5 kyphoplasty	_	_	_	_
2	77	M	Degenerative lumbar scoliosis	T10–L5 postero-lateral fusion	8	No	No	Osteoporosis	L5 stop	L5 screw pull-out	L4–L5 PSO, L4–L5 and L5–S1 TLIF, iliac bolts	S1 and iliac screw pull-out	Cementation around S1 and pelvic screws	_	_
3	52	F	L2–L3 degeneration; L3–L4 and L4–L5 DS	L2–L5 postero-lateral fusion	4	No	Yes	Smoker Alcohol	L5 stop	L5 pedicle screw pull-out, L4–L5 instability progression	T10-pelvis (S2AI screws); L5 PSO Schwab type 4	Iliac screw fracture	Double iliac screws	Iliac screw fracture, L5–S1 non-union	L5–S1 ALIF, iliosacral screws, satellite right rod
4	70	F	Lumbar Kyphoscoliosis and L2–L3, L3–L4 and L4–L5 DS	T9–L5 fusion; L3 PSO Schwab type 4, L3–L4 TLIF	9	Yes	N/A	_	L5 stop	Left L5 pedicle screw pull-out	L4–L5 and L5–S1 TLIF, iliac bolts	_	_	_	_
5	76	F	Lumbar kyphoscoliosis and L2–L3, L3–L4 and L4–L5 DS	T10–L5 fusion, L2–L3 decompression, TLIF L3L4	8	Yes	N/A	Parkinson’s disease, Obesity	_	L4–L5 stenosis	L4–L5 decompression	Bilateral pedicle screw pull-out/fracture	L5 PSO, L4–L5 TLIF, bilateral iliac fixation	_	_
6	68	F	Degenerative lumbar kyphoscoliosis	T10–S1 fusion, L3–L4 and L4–L5 decompression	9	No	No	Obesity	S1 stop, no iliac bolt	L5–S1 early kyphosis (DJK)	No revision yet	_	_	_	_
7	49	M	T10 fracture (T9–T11 fixation) post-traumatic kyphosis	T7–L1 fusion + PSO T9–T10	7	No	Yes	_	_	AO A.1 fracture of L3	L3 kyphoplasty	_	_	_	_
8	80	F	L1–L2, L2–L3 and L3–L4 stenosis	L1–L5 fusion	5	No	N/A	Obesity Diabetes	L5 stop	L4 screw pull-out; L4–L5 instability	L3–L4 TLIF, 2 level distal extension (S2)	_	_	_	_
9	59	M	Thoraco–lumbar kyphoscoliosis	T10–T11 dynamic stabilization, T11–S1 fusion, TLIF L2–L3, L3–L4 and L4–L5	8	Yes	No	Parkinson’s disease	S1 stop, no iliac bolt	S1 screw pull-out + L5–S1 instability	L5–S1 ALIF (only strut graft) + iliac bolts	L5–S1 non-union	L5–S1 ALIF with cage + rod change	Right Iliac Screw Fracture	Screw substitution
10	65	F	Lumbar degenerative kyphoscoliosis	T10–L5,TLIF L4L5	8	Yes	No	Osteoporosis	L5 stop	L5 fracture + L5–S1 stenosis	L5–S1 ALIF	ALIF L5 screw fracture + L5–S1 non-union	Not revised yet	_	_
11	20	M	Kyphoscoliosis (double major) Prader Willi	T2–L4 fusion	15	No	N/A	Obesity	L5 stop	L5 fracture	L4 PSO, iliac extension	_	_	_	_
12	62	F	L3–L4 and L4–L5 lumbar stenosis	L3–L5 decompression and posterolateral fusion	3	No	N/A	Osteoporosis Smoker	L5 stop	L5–S1 instability	T12-pelvis (iliosacral screws), L5–S1 TLIF	_	_	_	_
13	64	M	L3–L4 lumbar stenosis; L4–L5 DS	L3–L5 decompression and posterolateral fusion	3	No	Yes	_	L5 stop	L2–L3 and L5–S1 stenosis	L2–L3 fusion, L5–S1 decompression	L5–S1 iatrogenic spondylolisthesis	L5–S1 fusion left iliac fixation	_	_
14	64	F	L2–L5 stenosis	L2–L5 decompression and posterolateral fusion	4	No	Yes	Obesity, Smoker, Parkinson’s disease	L5 stop	L5 fracture, T8–T9 disc herniation, L1–L2 stenosis	T8–T9 decompression, L4–L5 PSO + T2-pelvis fusion, L4–L5 TLIF	_	_	_	_
15	70	F	Multilevel lumbar stenosis	T12–S1 decompression and posterolateral fusion	7	No	Yes	Obesity, Smoker	Sagittal imbalance Flat back	Screw pull-out L5 and S1	L5 PSO Schwab type 4,T10-pelvis fusion	_	_	_	_
16	78	M	Multilevel lumbar stenosis (L1–S1 fusion)	L1–L5 decompression and posterolateral fusion	5	No	N/A	Osteoporosis	_	Proximal screw pull-out (PJF)	L4 PSO + T8-pelvis fusion, L5–S1 TLIF	Iliac screw fractures	Iliosacral screws, L4–L5 and L5–S1 ALIF	L1 non-union (intercalary junctional failure)	5 Rod Construct
17	69	F	Multilevel lumbar DS	L3–S1 decompression	4	No	Yes	Parkinson’s disease	_	L5–S1 compression	T11-pelvis fusion, L3–L4 and L4–L5 LLIF	Pelvic fixation pull-out	Iliosacral screws, L4–L5 and L5–S1 ALIF	_	_
18	65	F	Multilevel lumbar stenosis L2–L5 with prior L2–L5 decompression/Thoracolumbar kyphosis	T10-pelvis fusion, L3–L4, L4–L5, and L5–S1 TLIFs	9	Yes	N/A	Obesity	_	Pelvic fixation pull-out	L4 PSO Schwab type 3, T8-pelvis fusion	Pelvic fixation pull-out	Iliosacral screws	_	_
19	69	M	Lumbar degenerative kyphoscoliosis	T10-pelvis fusion and L5–S1 TLIF	9	Yes	Yes	Osteoporosis	Gas in L2–L3, L3–L4, L4–L5 discs	Pelvic fixation pull-out	Pelvic revision with iliosacral screws				

DS: degenerative spondylolisthesis; IBD: interbody device at the most caudal level; RF: risk factors; PSO: pedicle subtraction osteotomy; S2AI: S2 alar iliac.

**Table 2:** Comparison of radiographic parameters during follow-up (SVA—sagittal vertical axis, PI—pelvic incidence, PT—pelvic tilt, SS—sacral slope, LL—lumbar lordosis).

	Pre Op Index	Post Op Index	*p*	Pre Operative 2	Post Operative 2	*p*	Pre Operative 3	Post Operative 3
SVA	60.43 ± 55.24	37.64 ± 34.4	0.211	78.52± 64.57	41 ± 45.22	0.065	139.05 ± 20.53	126 ± 12.72
PI	53.93 ± 19.9	53.14 ± 12.6	0.413	54.42 ± 22.16	52.94 ± 19.53	0.984	71.5 ± 23.33	68 ± 24.04
LL (L1S1)	21 ± 28.1	32.62 ± 34	0.1	0.5 ± 34.86	15.3 ± 48.3	0.226	34.33 ± 36.67	62.5 ± 3.53
PI-LL	30.79 ± 20.9 *	9.81 ± 11.7 *	0.013	31 ± 27.67	9.64 ± 18.04	0.0548	11.67 ± 11.71	5.5 ± 20.50
SS	21.1 ± 15.05	23.1 ± 16.4	0.378	13.5 ± 17.09 *	25.37 ± 16.13 *	0.022	28.67 ± 19.50	54.5 ± 16.26
PT	30 ± 14.2	24.4 ± 8.1	0.129	34.75 ± 17.17 *	22.06 ± 14.52 *	0.032	22 ± 19.67	13.05 ± 7.77

Pre Operative 2: before revision for first failure (Failure 1). Post Operative 2: after revision for first failure (Failure 1). Pre Operative 3: before revision for first failure (Failure 2). Post Operative 3: after revision for first failure (Failure 2). Asterisks (*) indicate statistical significance, *p* < 0.05.

## Data Availability

The original contributions presented in the study are included in the article, further inquiries can be directed to the corresponding author.

## References

[1] Arlet V., Aebi M. (2013). Junctional spinal disorders in operated adult spinal deformities: Present understanding and future perspectives. Eur. Spine J..

[2] Ames C.P., Scheer J.K., Lafage V., Smith J.S., Bess S., Berven S.H., Mundis G.M., Sethi R.K., Deinlein D.A., Coe J.D. (2016). Adult spinal deformity: Epidemiology, health impact, evaluation, and management. Spine Deform..

[3] Bridwell K.H., Lenke L.G., Cho S.K., Pahys J.M., Zebala L.P., Dorward I.G., Cho W., Baldus C., Hill B.W., Kang M.M. (2013). Proximal junctional kyphosis in primary adult deformity surgery: Evaluation of 20 degrees as a critical angle. Neurosurgery.

[4] Yasuda T., Hasegawa T., Yamato Y., Kobayashi S., Togawa D., Banno T., Arima H., Oe S., Matsuyama Y. (2016). Lumbosacral Junctional Failures After Long Spinal Fusion for Adult Spinal Deformity—Which Vertebra Is the Preferred Distal Instrumented Vertebra?. Spine Deform..

[5] Siambanes D., Mather S. (1998). Comparison of plain radiographs and CT scans in instrumented posterior lumbar interbody fusion. Orthopedics.

[6] Lee J.H., Lee J.-H., Park J.-W., Lee H.S. (2011). Fusion rates of a morselized local bone graft in polyetheretherketone cages in posterior lumbar interbody fusion by quantitative analysis using consecutive three-dimensional computed tomography scans. Spine J..

[7] Kwon B.K., Elgafy H., Keynan O., Fisher C.G., Boyd M.C., Paquette S.J., Dvorak M.F. (2006). Progressive junctional kyphosis at the caudal end of lumbar instrumented fusion: Etiology, predictors, and treatment. Spine.

[8] Denis F., Sun E.C., Winter R.B. (2009). Incidence and risk factors for proximal and distal junctional kyphosis following surgical treatment for Scheuermann kyphosis: Minimum five-year follow-up. Spine.

[9] Bouloussa H., Alzakri A., Ghailane S., Vergari C., Mazas S., Vital J.-M., Coudert P., Gille O. (2017). Is it safe to perform lumbar spine surgery on patients over eighty five?. Int. Orthop..

[10] Day L.M., DelSole E.M., Beaubrun B.M., Zhou P.L., Moon J.Y., Tishelman J.C., Vigdorchik J.M., Schwarzkopf R., Lafage R., Lafage V. (2018). Radiological severity of hip osteoarthritis in patients with adult spinal deformity: The effect on spinopelvic and lower extremity compensatory mechanisms. Eur. Spine J..

[11] Ameri E., Behtash H., Mobini B., Ghandhari H., Tari H.V., Khakinahad M. (2011). The prevalence of distal junctional kyphosis following posterior instrumentation and arthrodesis for adolescent idiopathic scoliosis. Acta Medica Iran..

[12] Schwab F.J., Blondel B., Bess S., Hostin R., Shaffrey C.I., Smith J.S., Boachie-Adjei O., Burton D.C., Akbarnia B.A., Mundis G.M. (2013). International Spine Study Group (ISSG). Radiographical spinopelvic parameters and disability in the setting of adult spinal deformity: A prospective multicenter analysis. Spine.

[13] Kim J.S., Phan K., Cheung Z.B., Lee N., Vargas L., Arvind V., Merrill R.K., Gidumal S., Di Capua J., Overley S. (2019). Surgical, radiographic, and patient-related risk factors for proximal junctional kyphosis: A meta-analysis. Glob. Spine J..

[14] Gottfried O.N., Daubs M.D., Patel A.A., Dailey A.T., Brodke D.S. (2009). Spinopelvic parameters in postfusion flatback deformity patients. Spine J..

[15] Shah A., Lemans J.V., Zavatsky J., Agarwal A., Kruyt M.C., Matsumoto K., Serhan H., Agarwal A., Goel V.K. (2019). Spinal balance/alignment—Clinical relevance and biomechanics. J. Biomech. Eng..

[16] Lowe T.G., Lenke L., Betz R., Newton P., Clements D., Haher T., Crawford A., Letko L., Wilson L.A. (2006). Distal junctional kyphosis of adolescent idiopathic thoracic curves following anterior or posterior instrumented fusion: Incidence, risk factors, and prevention. Spine.

[17] Richards B.S., Birch J.G., Herring J.A., Johnston C.E., Roach J.W. (1989). Frontal plane and sagittal plane balance following Cotrel-Dubousset instrumentation for idiopathic scoliosis. Spine.

[18] Ghasemi A., Stubig T., Nasto L.A., Ahmed M., Mehdian H. (2017). Distal junctional kyphosis in patients with Scheuermann’s disease: A retrospective radiographic analysis. Eur. Spine J..

[19] Terran J., Schwab F., Shaffrey C.I., Smith J.S., Devos P., Ames C.P., Fu K.-M.G., Burton D., Hostin R., Klineberg E. (2013). The SRS-Schwab adult spinal deformity classification: Assessment and clinical correlations based on a prospective operative and nonoperative cohort. Neurosurgery.

[20] Schwab F.J., Smith V.A., Biserni M., Gamez L., Farcy J.-P.C., Pagala M. (2002). Adult scoliosis: A quantitative radiographic and clinical analysis. Spine.

[21] Berjano P., Bassani R., Casero G., Sinigaglia A., Cecchinato R., Lamartina C. (2013). Failures and revisions in surgery for sagittal imbalance: Analysis of factors influencing failure. Eur. Spine J..

[22] Ghailane S., Pesenti S., Peltier E., Choufani E., Blondel B., Jouve J.L. (2017). Posterior elements disruption with hybrid constructs in AIS patients: Is there an impact on proximal junctional kyphosis?. Arch. Orthop. Trauma Surg..

[23] Nicholls F.H., Bae J., Theologis A.A., Eksi M.S., Ames C.P., Berven S.H., Burch S., Tay B.K., Deviren V. (2017). Factors associated with the development of and revision for proximal junctional kyphosis in 440 consecutive adult spinal deformity patients. Spine.

[24] Yang J., Andras L.M., Broom A.M., Gonsalves N.R., Barrett K.K., Georgiadis A.G., Flynn J.M., Tolo V.T., Skaggs D.L. (2018). Preventing distal junctional kyphosis by applying the stable sagittal vertebra concept to selective thoracic fusion in adolescent idiopathic scoliosis. Spine Deform..

[25] Cho K.-J., Suk S.-I., Park S.-R., Kim J.-H., Choi S.-W., Yoon Y.-H., Won M.-H. (2009). Arthrodesis to L5 versus S1 in long instrumentation and fusion for degenerative lumbar scoliosis. Eur. Spine J..

[26] Hu S., Berven S. (2006). Preparing the Adult Deformity Patient for Spinal Surgery. Spine.

[27] McDonnell J.M., Ahern D.P., Wagner S.C., Morrissey P.B., Kaye I.D., Sebastian A.S., Butler J.S. (2021). A Systematic Review of Risk Factors Associated with Distal Junctional Failure in Adult Spinal Deformity Surgery. Clin. Spine Surg..

[28] McDonnell J.M., Evans S.R., Ahern D.P., Cunniffe G., Kepler C., Vaccaro A., Kaye I.D., Morrissey P.B., Wagner S.C., Sebastian A. (2022). Risk factors for distal junctional failure in long-construct instrumentation for adult spinal deformity. Eur. Spine J..

[29] Montanari S., Griffoni C., Cristofolini L., Girolami M., Gasbarrini A., Barbanti Bròdano G. (2023). Correlation Between Sagittal Balance and Mechanical Distal Junctional Failure in Degenerative Pathology of the Spine: A Retrospective Analysis. Glob. Spine J..

